# Prevalence of domestic violence during pregnancy and related risk factors: a cross-sectional study in southern Sweden

**DOI:** 10.1186/1472-6874-14-63

**Published:** 2014-05-01

**Authors:** Hafrún Finnbogadóttir, Anna-Karin Dykes, Christine Wann-Hansson

**Affiliations:** 1 Faculty of Health and Society, Department of Care Science, Malmö University, Malmö, Sweden; 2 Department of Health Sciences, Medical Faculty, Lund University, Lund, Sweden; 3 The Swedish Institute of Health Sciences (Vårdalinstitutet), Lund University, Lund, Sweden

**Keywords:** Domestic violence, Pregnancy, Prevalence, Risk factors, Depression

## Abstract

**Background:**

Domestic violence during pregnancy is a serious public health issue which threatens maternal and foetal health outcomes. The *aim* of the study was to explore prevalence of domestic violence among pregnant women in southern Sweden (Scania) and to explore associations with background factors, as symptoms of depression and sense of coherence.

**Methods:**

This study has a cross-sectional design and is the first part of a longitudinal, cohort study. Inclusion criteria were women ≥ 18 years, registered at antenatal care when pregnant and who understand and write Swedish or English. Questionnaires were collected prospectively at seventeen antenatal care receptions situated in the two cities and six smaller municipalities in Scania. Statistical analyses were done using descriptive statistics, chi-square tests, bivariate logistic regression and multiple regression with Odds ratios (OR) and 95% confidence intervals (95% CI).

**Results:**

Study sample included 1939 women. History of violence was reported by 39.5% (n =761) women. Significant differences were obtained between the groups with or without history of violence regarding being single/living apart, unemployment, financial distress, smoking/snuffing, unintended pregnancy as well as history of miscarriage/legalised abortion (p < 0.001). Experience of domestic violence during pregnancy regardless of type or level of abuse was 1.0% (n = 18); history of physical abuse by actual intimate partner was 2.2% (n = 42). History of violence was the strongest risk factor associated with domestic violence during pregnancy, where all women (n = 18) exposed reported history of violence (p < 0.001). Several symptoms of depression (adjusted for low socio-economic status, miscarriage/abortion, single/living apart, lack of sleep, unemployment, age and parity) were associated with a 7.0 fold risk of domestic violence during pregnancy (OR 7.0; 95% CI: 1.9-26.3).

**Conclusions:**

The reported prevalence of domestic violence during pregnancy in southwest Sweden is low. However, a considerable proportion of women reported history of living in a violent relationship. Both history of violence and the presence of several depressive symptoms detected in early pregnancy may indicate that the woman also is exposed to domestic violence during pregnancy. Increased attention to this vulnerable group of women is needed to improve maternal and child health.

## Background

Domestic violence (DV) during pregnancy is a serious public health issue which threatens maternal and foetal health outcomes [1-7]. DV is defined according to World Health Organisation (WHO) as psychological/emotional, physical, or sexual violence, or threats of physical or sexual violence that are inflicted on a woman by a family member: an intimate male partner, marital/cohabiting partner, parents, siblings, or a person very well known within the family or a significant other (i.e. former partner) when such violence often takes place in the home [8].The prevalence of DV against pregnant women varies widely in the literature, ranging from 1.2 to 66% [2]. This variation is probably attributable to differences across studies in sampled populations, as well as differences in methodologies, definitions, and cultural aspects that make it difficult to compare the results [2,9]. The prevalence regarding intimate partner violence (IPV) during pregnancy has been demonstrated in the first global report of internationally comparable data on populations from 19 countries, ranged between 2.0% and 13.5% [10]. A recently published meta-analysis of 92 independent studies concerning prevalence and risk factors associated with DV among pregnant women showed an average prevalence of emotional abuse of 28.4%, and prevalence rates of physical abuse and sexual abuse were 13.8% and 8.0%, respectively [11]. Further, the overall prevalence of DV during pregnancy in less developed countries is higher (27.7%) than that in developed countries (13.3%) [11]. Most of the violence against women occurs at home; thus women are more at risk of violence from an intimate partner than from any other type of perpetrator [12].

A meta-analysis of 55 independent studies found that the strongest predictor of DV among pregnant women was experience of abuse before pregnancy [11]. Pregnant women whose partners previously abused them had four times greater odds of being abused during pregnancy than those women who had no history of violence. Other risk factors identified for DV among pregnant women were single marital status, lower education, low socioeconomic status, alcohol abuse (above all by the perpetrator), and unintended or unwanted pregnancy [11]. IPV is a strong risk factor for unintended pregnancy and abortion across variety of settings worldwide [13], and women undergoing repeated induced abortion are more likely to have a history of physical abuse by a male partner or a history of sexual abuse [13-15]. High levels of symptoms of mental disorders such as depression, anxiety and post-traumatic stress disorder during the perinatal period are also significantly associated with experience of DV both during lifetime and pregnancy [16].

DV during pregnancy also confers a risk for the unborn child. Thus, a systematic review of thirty studies showed that abused pregnant women are 1.5 times more likely to deliver a low birth-weight baby and almost 1.5 times more likely to have preterm births [7]. Moreover, ablation placenta, uterus rupture, [17,18] foetal trauma [18,19] or foetal death [19-21] have also been reported. The most extreme consequence of violence during pregnancy is femicide (homicide of females) and most likely by a current or former intimate partner [22].

In previous Swedish prevalence studies of physical or sexual abuse during pregnancy the prevalence varied between 1.3% and 11% [23-25]. However, these studies were conducted almost two decades ago, and due to continuous societal changes it is important to obtain more current prevalence rates. Further, the Swedish National Council for Crime Prevention has reported increasing numbers of abused women during the last two decades, with an increase of 1% during 2012 and primarily increases in single mothers and women in the workforce. The increasing figures can partly be explained by changes in legislation in the beginning of the 1980s such that abused women could no longer withdraw already submitted written reports of abuse [26]. Also, several studies from different regions in the country are required in order to be able to understand the entire population in the increasingly multicultural society of Sweden, as well as to allocate resources to those regions that might have higher prevalence rates of DV. Finally, results from a survey concerning DV during pregnancy would highlight the problem and hopefully increase awareness and action for identification and prevention. The *aim* of the study was to explore the prevalence of DV among pregnant women in southwest Sweden in the region of Scania and to identify possible differences between groups with or without a history of violence. A further aim was to explore associations between DV and potential risk factors such as; i) socio-demographic background variables ii) maternal characteristics iii) high risk health behaviour iv) self-reported health-status and sleep as well as symptoms of depression, and v) sense of coherence.

## Methods

This study has a cross-sectional design and is the first part of a longitudinal cohort study. According to the WHO’s ethical and safety recommendations for research into DV against women it is important that the survey on violence is framed in a different way, and also that the woman is fully informed about the nature of the questions [27]. Our study is framed as “*Pregnant women and new mothers’ health and life experience”* were *‘life experience’* covers experienced violence*.* Pregnant women who fulfilled the inclusion criteria for the study were consecutively recruited during their first visit at Antenatal Care (ANC) for study participation. *Inclusion criteria* were women ≥ 18 years, registered at ANC when pregnant and who understood and could write Swedish or English. A power analysis indicated that at least 2000 participants were needed to detect with 98% certainty at least 2.5% prevalence of DV.

### ANC’s services in Sweden

In Sweden the ANC services are included in the overall health insurance system, free of charge (inclusive private care facilities) and available all over the country. Since the autumn 2011, private care facilities have increased in number, and women have the right to choose the type of care and midwife by herself. Midwives have the main responsibility for the normal pregnancy, and the father-to-be is also welcome to attend ANC visits. According to Swedish health care reports, almost 100% of pregnant women utilise their right to ANC services [28].

### Settings and participants

The geographical area of Scania in southwest Sweden is characterised by multicultural diversity. Initially 26 ANCs in the area, a multicultural city with > 300 000 inhabitants, a university city with > 110 000 thousand inhabitants and surrounding municipalities were asked to participate in this study, among which nine ANCs declined. Four public ANC’s in the municipality’s area and five privately driven ANC’s in the multicultural city declined to participate in the study due to high work load, or a new organization. The population includes all registered pregnant women at 17 ANCs situated in the multicultural city (n = 7), the University City (n = 4) and smaller municipalities (n = 6). One ANC providing specialised care for complicated pregnancies such as women with diabetes and one unique activity group for women with history of drug abuse in need of extra support were also included. Two of the ANCs in the multicultural city, one ANC in the University City and one ANC in the municipalities are private care facilities. Most of the women in the sample would presumably give birth at the regional university hospital, which has two separate delivery departments, with an approximately birth rate of 8000–9000 deliveries per year.

### Recruitment

Data were collected prospectively between March 2012 and September 2013. Approximately 80 midwives performed the recruitment. Prior to the study all recruiting midwives were personally informed about the study design by the first author (HF). At every participating ANC maximally 24 to 29 questionnaires were distributed to each midwife. The pregnant women were invited to participate during their first visit to ANC, during the 6-8th week of pregnancy or at the visit when registered at the ANC during gestational weeks 11–13. If the midwife missed the opportunity to recruit the woman at these time periods, she was given the opportunity to recruit that woman at the latest during gestational week 25. If the woman had been delayed in registration at the ANC, the midwife was still encouraged to recruit her. The pregnant women received individually verbal and written information about the study by their midwife and were invited to answer the questionnaire in a private place at the ANC facility (possibilities for privacy varied between the facilities). After giving written informed consent, they received the questionnaire. The participant placed the completed questionnaire in a sealed envelope together with the signed consent form, which was similarly placed in a smaller sealed envelope and handed them over to the recruiting midwife. The woman was promised confidentiality and it was completely up to her if she disclosed to her midwife that she was living in a violent relationship. All answered questionnaires were kept in a safe place until they were collected every third week by the first author (HF) who gave each questionnaire (participant) a unique code. Both participants and recruiting midwives had the possibility to e-mail or call the first author whenever they wished. To facilitate the recruitment when the women were accompanied by their partner, simultaneously the partner was invited to take part in another study completely independent from the present study, *Fathers to-be and new fathers’/partners’, health and lifestyle*. In the waiting room there were two different posters with information about the studies.

### Questionnaire

All data were based on a self-administrated questionnaire with 122 questions that took approximately 15–30 minutes to answer, depending on the individual.

### NorVold Abuse Questionnaire (NorAQ)

The main instrument was the NorAQ, constructed and validated in Nordic countries [29]. This instrument measures emotional, physical and sexual abuse as a child (<18 years) and as an adult (≥18 years), and also includes a question about the age when first subjected to abuse. Further, a yes/no question about experience of abuse during the past 12 months is included, followed by the question “by whom”, with eight alternatives and the possibility of *a write-in alternative.* All answer alternatives (‘boxes to tick in’) are followed by the alternative “by male” or “by female”. The abuse variables in NorAQ have previously shown good reliability, validity and specificity [29]. All questions about abuse from the NorAQ questionnaire were administered in their original format in order to maintain the instrument’s reliability, validity and specificity. Further, the questionnaire also included one question concerning health and one concerning sleep from the original NorAQ. The health question “How do you feel your health has been, generally speaking, for the last 12 months?” had the following four alternatives: i) very good, ii) rather good, iii) rather poor, iiii) very poor; sleep question “During the last 12 months, have you suffered from insomnia to such an extent that you have had problems coping with your daily life?” had the following four alternatives: i) No, ii) yes but rarely, iii) yes sometimes, iiii) yes often. In addition, the questionnaire contained questions validated and applied in the Nordic abuse study [30] relating to health and socio-demographic background.

### Additionally questions to the questionnaire

One modified question was used from the *Abuse Assessment Screen* (AAS), “*Have you been exposed to abuse during current pregnancy?”* in order to investigate emotional, physical, and sexual abuse (yes/no, if yes by whom). One question concerned private economy: “*If you received an unexpected bill of 20.000 SEK, (approximately USD 3000 or 1875 GBP or 2243 EUR) how easy would it be for you to pay within a week?” *[31] Choices were: i) no problem, ii) pretty hard, iii) very hard.

### Sense of coherence scale (SOC-13)

Views on life, stress management and the use of one’s own resources to maintain and improve health were measured by a short form of the SOC-13 [32]. The SOC-scale instrument is reliable, valid and cross-culturally applicable with acceptable face validity [33]*.* Strong SOC (high score) is a significant predictor of good health [34].

### Edinburgh Postnatal Depression Scale (EPDS)

Symptoms of depression were assessed using the EPDS, an instrument covering common symptoms of depression and that is designed to screen for risk of depression during the postnatal period [35], but can also be used during pregnancy (EDS) [36]. The instrument EPDS has a satisfactory sensitivity (85%) and specificity (77%) [35], and has been validated in a Swedish community sample against criteria for major depression, according to the Diagnostic and Statistical Manual of Mental Disorders (DSM-III-R) [37]. Also, the EDS has been validated for the detection of depressive symptoms during pregnancy with an optimal cut-off at ≥ 13 and indicates qualification for a diagnosis of probable depression (DSM-IV) [36]. The instrument has a sensitivity of 77% according to DSM-IV criteria and a specificity of 94%. The current study used the EDS full scale with 10 items on a four point scale from 0–3 (high scores = more symptoms of depression).

### Alcohol Use Disorders Identification Test (AUDIT)

Finally, one question from the AUDIT was used for the detection of harmful alcohol consumption [38]. The question, which is the first item in the AUDIT, concerns the frequency of drinking alcohol. The answer alternatives were: ‘never’ or the amount of beverage consumption.

### Classification of the variables

*Age* was classified as 18–25, 26–34 and ≥ 35 years. *Country of origin* was classified as born in Sweden, in another Nordic country or in other countries. *Language* was dichotomised as Swedish language or foreign language spoken at home. *Educational status* was classified as compulsory school or less, high school or less, or university. *Cohabiting status* was classified as single, living apart, or common law spouse/married. *Employment status* was dichotomised as employed (including parental leave and studying) or unemployed (including long illness). *Financial distress* was dichotomised as “no” (no problem) or “yes” (serious financial distress).

Maternal characteristics concerning *body mass index* (BMI) were calculated from maternal weight and height before the pregnancy and classified according to WHO’s definition [39] as underweight (<18.5), normal weight (18.50- 24.99), overweight (≥25- 29.99), and obese (≥30). *Smoking* was dichotomised as “yes” (if the woman was a daily smoker or smoking at some point during pregnancy) and “no” (never smoked or ceased before pregnancy). *Snuffing* was dichotomised as “yes” (if the woman was a daily user of snuff or snuffing at some point during pregnancy) and “no” (never snuffed or ceased before pregnancy). *Use of alcohol* was dichotomised as “yes” or “no”. *Unintended pregnancy was dichotomised as* “yes” or “no”. *Abortion/miscarriage* was classified as “no”, “miscarriage”, “abortion” or both “miscarriage/abortion”.

### Definitions

The study uses Swahnberg et al.’s [29] definitions for severity of abuse, classified as mild, moderate or severe and also type of abuse. *Mild emotional abuse* is the experience of being systematically and persistently repressed, degraded or humiliated. *Moderate emotional abuse* is the experience of being systematically and by threat or force restricted with regard to contacts with others or subjected to total control concerning what one may and may not do. *Severe emotional abuse* is the experience of living in fear due to systematic and persistent threats by someone close.

*Mild physical abuse* is being hit, smacked in the face or held in involuntary restraint. *Moderate physical abuse* is being hit with the fist(s) or with a hard object, being kicked, violently pushed, or beaten, or similar experiences. *Severe physical abuse* is being exposed to life threatening experiences, such as attempted strangulation, being confronted by a weapon or knife, or any other similar act.

*Mild sexual abuse* (with no genital act) is being touched on parts of the body other than the genitals in a sexual way against one’s will or being forced to touch other parts of another person’s body in a sexual way. Further, *mild sexual abuse* (emotional or sexual humiliation) is the experience of being forced to watch a pornographic film, to participate in a pornographic film or similar, being forced to show one’s body naked or to look at someone else’s naked body. *Moderate sexual abuse* (genital contact) is the experience of being touched on the genitals against one’s will, being forced to satisfy him/herself sexually, or forced to touch another person’s genitals. *Severe sexual abuse* (penetration) is forced penetration of the penis into the vagina, mouth or rectum, or forced penetration or attempted penetration by an object or other part of the body into the vagina, mouth or rectum [29].

*History of violence* is defined as lifetime experience of emotional, physical or sexual abuse, occurring during childhood (<18 years), adulthood (≥18 years) or both, regardless of the level of abuse or the perpetrator’s identity, in accordance with the operationalization of the questions in the NorAQ [29].

### Ethical considerations

As recommended by the Declaration of Helsinki [40], the likelihood of benefits from the current research was considered. Research on violence against women during pregnancy raises important ethical and methodological challenges in addition to those raised by any other type of research on human subjects [27]. Therefore, the current study was conducted in accordance with the WHO’s ethical and safety recommendations for research on DV against women [27]. Approval was provided from the Regional Ethical Review Board in Southern Sweden (Dnr: 640/2008).

### Statistical methods

Descriptive statistics were used to show prevalence and severity of lifetime experience of any type and level of abuse (Table 1). Chi-square analysis was used to investigate differences in socio-demographic and maternal characteristics between women with and without reported ‘history of violence’ (Tables 2 and 3). OR and 95% CI were calculated for the crude associations between possible risk factors and ‘DV during pregnancy’, with ‘DV during pregnancy’ as a dependent variable for bivariate logistic regression. *Age* was dichotomised as 18–34 or ≥ 35 years, educational status as high school or less versus university, *language* as foreign language spoken at home or Swedish (solely), *cohabiting status* as single/living apart or cohabiting with spouse/married, and *smoking and/or snuffing* as “yes” versus “no”. *BMI* was dichotomised as under-/normal weight or overweight/obese, *miscarriage or abortion history* as miscarriages/abortions versus solely abortion, miscarriages or not at all, *self-reported health* as poor health versus rather good health, “*lack of sleep* versus *adequate sleep”*. For the purpose of bivariate logistic regression, a variable for depression was computed on the basis of EDS scores, i.e. symptoms of depression during pregnancy, whereby an optimal cut-off of ≥ 13 was chosen as representing presence of symptoms of depression [36]. The EDS score was computed only for those responding to all ten questions (missing = 62). In order to analyse the association between SOC score and exposure to ‘DV during pregnancy’, the SOC-scale was dichotomised, utilizing the first quartile of the distribution as a cut-off value (SOC ≤ 64 and SOC >64) [41]. The SOC score was only computed for those responding to all thirteen items (missing = 101). Multiple logistic regression was performed in order to evaluate the influence of variables that were significant in the bivariate logistic regression with ‘DV during pregnancy’ as a dependent variable; the multiple logistic regression analyses were thus step-wise adjusted (Forward selection) for EDS ≥ 13, SOC Low score, miscarriage/abortion, single/living apart, lack of sleep, unemployment and also age and parity. Statistical significance was accepted at p < 0.05. Statistical analyses were performed using the Statistical Package for Social Sciences (SPSS) version 21.0 for Windows.

**Table 1:** Type and severity of abuse: lifetime and during pregnancy (N = 1939)

**Type and severity of abuse**	***Missing***	**History of violence**^**a**^	**During pregnancy**^**b**^
	**n**	**n (%)**	**n (%)**
	11*	**761 (39.5)**	**29 (1.5)**
**Lifetime emotional abuse**	20	374 (19.5)	20 (1.0)
*Mild*	36	307 (16.1)	
*Moderate*	28	187 (9.8)	
*Severe*	28	203 (10.6)	
**Experienced emotional abuse first time**	18		
Age < 18 years		208 (58.4)	
Age ≥ 18 years		148 (41.6)	
**Any emotional abuse past year**^**c**^	5		
Yes		61 (16.5)	
No		308 (83.5)	
**Lifetime physical abuse**	24	561 (29.3)	7 (0.4)
*Mild*	53	529 (28.0)	
*Moderate*	41	203 (10.7)	
*Severe*	32	127 (6.7)	
**Experienced physical abuse first time**	41		
Age < 18 years		355 (68.3)	
Age ≥ 18 years		167 (31.7)	
**Any physical abuse past year**^**c**^	20		
Yes		36 (6.7)	
No		505 (93.3)	
**Lifetime sexual abuse**	20	302 (15.7)	2 (0.1)
*Mild*^*1*^	33	212 (11.1)	
*Mild*^*2*^	34	144 (7.6)	
*Moderate*	45	208 (11.0)	
*Severe*	53	49 (2.6)	
**Experienced sexual abuse first time**	12		
Age < 18 years		196 (67.6)	
Age ≥ 18 years		94 (32.4)	
**Any sexual abuse past year**^**c**^	7		
Yes		2 (0.7)	
No		293 (99.3)	

*Did not answer the questions about abuse, ^a^Any type of abuse during lifetime, ^b^Self-reported abuse during pregnancy irrespective of the perpetrator, ^c^Any type of abuse experienced past 12 months, ^1^Emotional or sexual humiliation, ^2^No genital contact.

**Table 2:** Distribution of socio-demographic background factors at recruitment to the study (N = 1939)

**Characteristics**	**Total**	**History of violence**^**a**^		**P**
		**No**	**Yes**	
	**n (%)**	**n (%)**	**n (%)**	**OR, 95% CI**
	**1928 (99.4)**	**1167 (60.5)**	**761 (39.5)**	
Missing*	11 (0.6)			
**Age, years**				NS
18-25	339 (17.5)	206 (17.8)	133 (17.8)	
26-34	1211 (62.5)	750 (64.9)	461 (61.6)	
≥ 35	354 (18.2)	200 (17.3)	154 (20.6)	
*Missing*	35 (1.8)			
**Country of origin**				NS
Sweden	1545 (79.6)	923 (79.2)	622 (81.8)	
Nordic countries	47 (2.5)	27 (2.3)	20 (2.6)	
Other countries	334 (17.2)	216 (18.5)	118 (15.5)	
*Missing*	13 (0.7)			
**Language**				NS
Swedish	1461(75.3)	871 (74.9)	590 (77.7)	
Foreign language	461 (23.8)	292 (25.1)	169 (22.3)	
*Missing*	17 (0.9)			
**Educational status**				NS
Compulsory school or less	60 (3.1)	29 (2.5)	31 (4.1)	
High school or less	576 (29.7)	338 (29.0)	238 (31.3)	
University	1291 (66.6)	799 (68.5)	492 (64.7)	
*Missing*	12 (0.6)			
**Cohabiting status**				< 0.001 1.6 (1.4-1.9)
Single	55 (2.8)	22 (2.0)	33 (4.4)	
Living apart	51 (2.6)	19 (1.7)	32 (4.3)	
Common law spouse/married	1763 (91.0)	1085 (96.4)	678 (91.3)	
*Missing*	70 (3.6)			
**Employment status**				< 0.001 2.2 (1.5-3.3)
Employed	1820 (93.9)	1121 (96.1)	699 (91.9)	
Unemployed	107 (5.5)	45 (3.9)	62 (8.1)	
Missing	12 (0.6)			
**Financial distress**				< 0.001 1.5 (1.2-1.8)
No	1004 (51.8)	653 (56.0)	351 (46.2)	
Yes	922 (47.5)	513 (44.0)	409 (53.8)	
*Missing*	13 (0.7)			

Statistical significance is accepted at p < 0.05, two-tailed.

Chi-square analysis is used.

^a^Has reported lifetime experience of emotional, physical or sexual abuse.

*Did not answer the questions about abuse.

**Table 3:** Overview of maternal characteristics and high risk health behavior at recruitment (N = 1939)

**Characteristics**	**Total**	**History of violence**^**a**^		
		**No**	**Yes**	**P**
	**n (%)**	**n (%)**	**n (%)**	
**Total n (%)**	**1928 (99.4)**	**1167 (60.5)**	**761 (39.5)**	**OR, 95% CI**^**b**^
Missing*	11 (0.6)			
**Parity**				NS
Primiparae	817 (42.1)	480 (44.9)	337 (47.3)	
Multiparae	966 (49.9)	590 (55.1)	376 (52.7)	
*Missing*	156 (8.0)			
**BMI**				NS
Underweight	79 (4.1)	51 (4.5)	28 (3.8)	
Normal weight	1289 (66.5)	789 (70.3)	500 (68.3)	
Overweight	232 (12.0)	198 (17.6)	134 (18.3)	
Obese	154 (4.3)	84 (7.5)	70 (9.6)	
*Missing*	85 (4.4)			
**Smoking**				<0.001 2.0 (1.5-2.5)
No	1575 (81.2)	991 (87.9)	584 (78.6)	
Yes	296 (31.5)	137 (12.1)	159 (21.4)	
*Missing*	68 (3.5)			
**Snuffing**				<0.001 2.7 (1.7-4.3)
No	1786 (92.1)	1096 (97.2)	690 (92.9)	
Yes	84 (4.3)	31 (2.8)	53 (7.1)	
*Missing*	69 (3.6)			
**Use of alcohol**				NS
No	878 (45.3)	528 (47.0)	350 (47.5)	
Yes	982 (50.6)	595 (53.0)	387 (52.5)	
*Missing*	79 (4.1)			
**Unintended pregnancy**				<0.001 1.8 (1.4-2.3)
No	1569 (80.9)	991 (85.9)	578 (76.9)	
Yes	336 (17.3)	162 (14.1)	174 (23.1)	
*Missing*	34 (1.8)			
**Abortion/miscarriage**				<0.001
No	1125 (58.0)	742 (65.3)	383 (51.8)	
Miscarriage	342 (17.7)	209 (18.4)	133 (18.0)	
Abortion	286 (14.8)	133 (11.7)	153 (20.7)	
Miscarriage/abortion	123 (6.3)	53 (4.7)	70 (9.5)	
*Missing*	63 (3.2)			

Statistical significance is accepted at p < 0.05, two-tailed. Chi-square analysis is used.

^a^Has reported lifetime experience of emotional, physical or sexual abuse,

^b^If the groups were ≥ 4, OR with CI not calculated.

*Did not answer the questions about abuse.

## Results

**Figure 1:**
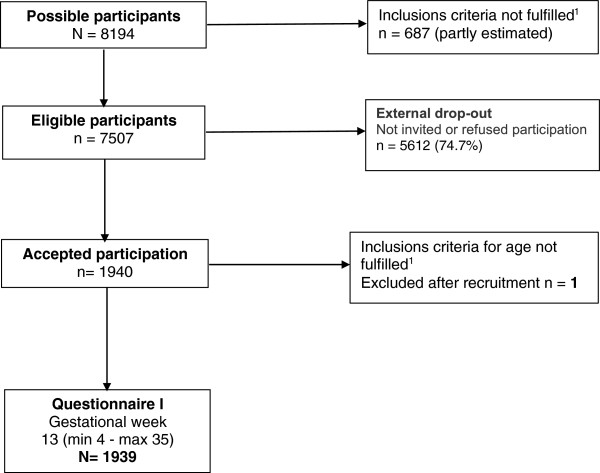
**Flowchart over recruitment to the study.** The study population includes all registered pregnant women during the recruitment time n = 8194 at the 17 attending ANCs. Of those, 687 women did not fulfill the inclusions criteria for age or sufficient reading and writing skills in Swedish or English leaving 7507 women as eligible participants. External drop-out constitutes of women not invited to take part in the study or those who refused participation leaving 1940 participants. One of those did not fulfill the inclusion criteria for age (≥18 years) and was therefore excluded.

In total 1940 women accepted participation in the study. One woman was excluded because of age ≤ 18 years (Figure 1), leaving 1939 women primarily recruited during gestational week 13 (mean 12.84, SD 5.11, min 4- max 35). The distribution of the participants was: multicultural city, 51.9% (n = 1006), University City 22.3% (n = 433) and surrounding municipalities 25.8% (n = 500). Almost 80% had Sweden as a country of origin and the remaining participants were born in 93 foreign countries. Reported ‘DV during pregnancy’, regardless of type or level of abuse, was 1.0% (n = 18) in the entire cohort. Greater proportion of women born outside the Nordic countries compared to the native of Sweden reported DV during pregnancy (RR, 2.4). In the total cohort 39.5% (n =761) of the women reported experience of ‘history of violence’ with eleven answers missing (Table 1). Among the eleven cases with missing answers, there was a greater percentage of women who were foreign-born, who spoke foreign languages at home, and who were low educated.

### DV during pregnancy and abuse committed by intimate partner (solely)

DV by actual intimate partner in terms of lifetime emotional abuse was 0.8% (n =16) and seven of these reported ‘DV during pregnancy’. Reported DV by actual intimate partner in terms of lifetime physical abuse was 2.2% (n = 42) and seven of these reported ‘DV during pregnancy’. Reported DV by actual intimate partner in terms of lifetime sexual abuse was 0.2% (n =4) and two of these reported ‘DV during pregnancy’.

### DV and the perpetrator

Of those 19.5% (n = 374) women who reported lifetime emotional abuse (Table 1), 66.3% (n = 248) were exposed to DV and the perpetrator was male in all cases and in six cases also female (figures not offered in Table 1). Among the 29.3% (n = 561) women who reported lifetime physical abuse (Table 1), 74.2% (n = 416) were exposed to DV and the perpetrators were male in all cases but one, and in 28 cases females were also involved (figures not offered in Table 1). Among those 15.7% (n = 302) women who reported lifetime sexual abuse (Table 1), 37.1% (n = 112) were exposed to DV and the perpetrators were male in all cases, and in one case also female (figures not offered in Table 1).

### Experience of a history of violence

Table 1 provides prevalence and severity of lifetime experience of emotional 19.5% (n = 374), physical 29.3% (n = 561) and sexual 15.7% (n = 302) abuse as well as experienced abuse during pregnancy 1.5% (n = 29) solely. Emotional abuse during current pregnancy was experienced by 1% (n = 20), physical abuse by 0.4% (n = 7) and sexual abuse by 0.1% (n =2). Of those women who reported ‘history of violence’, 16.5% (n = 61) had experienced emotional abuse, 6.7% (n = 36) physical abuse and 0.7% (n = 2) sexual abuse during the past year (Table 1).

### Differences between groups with or without a history of violence

Table 2 shows the distribution of the socio-demographic factors for the total cohort (n =1939) of women with or without experience of a “history of violence”. Statistical differences were found between the groups with regards to cohabitation, employment- and financial distress (p = 0.001). Further, Table 3 shows the results regarding maternal characteristics and high risk health behaviour for women with or without experience of a ‘history of violence’. There were statistical difference between the groups regarding smoking and snuffing, unintended pregnancies and experience of legalised abortion or having had both a miscarriage and legalised abortion (p < 0.001).

### Association between possible risk factors and exposure to DV during pregnancy

The strongest risk factor for DV during pregnancy was ‘history of violence’, whereby all women (n = 18) exposed to ‘DV during pregnancy’ also had reported ‘history of violence’ (p < 0.001). Unemployed women were 5.1 times more likely to report being exposed to ‘DV during pregnancy’ (p < 0.002). Women who were single or living apart were 6.9 times more likely to be exposed to ‘DV during pregnancy’ (p < 0.001). Further, women having a history of miscarriages and abortions were 7.6 times more likely to be exposed to ‘DV during pregnancy’ (p < 0.001). Those who reported lack of sleep during the past year were 4.7 times more likely to be exposed to ‘DV during pregnancy’ (p = 0.001). Women having EDS score ≥ 13 indicating presence of several symptoms of depression were 13.4 times more likely to be exposed to ‘DV during pregnancy’ (p < 0.001). Finally, women having low score on SOC indicating inability to use their own resources to maintain and improve their health in stressful situations were 9.1 more likely to be exposed to ‘DV during pregnancy’ (p < 0.001) (Table 4).

**Table 4:** **Association between possible risk factors and exposure to *DV during pregnancy *(N = 1939)**

**Independent variabel**	**n%**	**DV during pregnancy n (%)**	**OR 95% CI**	**P-value (two-tailed)**
History of violence^1^	745	18 (2.4)	-	<0.001
Age ≥ 35	351	6 (1.7)	2.6 (0.9-7.3)	NS
Multiparae	949	12 (1.3)	0.6 (0.2-1.6)	NS
Low educational status	616	7 (1.1)	1.3 (0.5-3.4)	NS
Unemployed	103	4 (3.9)	5.1 (1.7-15.9)	0.002
Foreign language	442	6 (1.4)	1.6 (0.61-4.40)	NS
Single/living apart	101	5 (5.0)	6.9 (2.4 -19.7)	0.001
Financial distress	896	12 (1.3)	2.2 (0.8-6.0)	NS
Alcohol consumption	971	8 (0.8)	0.7 (0.3-1.8)	NS
Smoking/snuffing	345	5 (1.4)	1.5 (0.6 - 4.7)	NS
Overweight/obese	478	6 (1.3)	1.4 (0.5-3.8)	NS
Unintended pregnancy	331	5 (1.5)	1.8 (0.6-5.1)	NS
Miscarriage/abortion	119	6 (5.0)	7.6 (2.8 - 20.6)	<0.001
Self-reported poor health	109	2 (1.8)	2.0 (0.5-9.0)	NS
Lack of sleep	145	5 (3.4)	4.7 (1.7 - 13.5)	0.001
EDS ≥ 13	166	10 (6.0))	13.4 (5.2- 34.4)	<0.001
SOC Low score	454	12 (2.6)	9.1 (2.9-28.5)	<0.001

Statistical significant is accepted at p < 0.05.

^1^All (n = 18) reported history of violence and therefore OR with 95% CI not showed.

When the analyses were controlled for low SOC score, miscarriage/abortion, single/living apart, lack of sleep, unemployment (significant in the bivariate analysis), age and parity, only EDS ≥ 13 remained significant (p < 0.004) and had 7.0 fold risk associated with ‘DV during pregnancy’. Marginal associations were also found between ‘DV during pregnancy’ and miscarriage/abortion (p = 0.053), low SOC-score (p = 0.075) and age ≥ 35 years (p = 0.097) (Table 5).

**Table 5:** Association between possible risk factors and exposure to DV during pregnancy (n = 18) presented by OR with 95% CI

**Variables**	**Model I**	**Model II**	**Model III**	**Model IV**	**Model V**	**Model VI**	**Model VII**	**Model VIII**
EDS ≥ 13^a^	13.9 (4.8-40.7)	7.0 (2.0-25.2)	6.2 (1.7-22.4)	6.1 (1.7-22.3)	6.9 (1.9-24.9)	6.8 (1.9-24.9)	7.1 (1.9-26.3)	7.0 (1.9-26.3)
Low score SOC^b^		3.3 (0.8-13.2)	3.3 (0.8-13.1)	3.3 (0.8-13.1)	3.6 (0.9-14.3)	3.4 (0.8-13.6)	3.6 (0.9-14.7)	3.6 (0.9-14.9)
Miscarriage/abortion^c^			4.2 (1.2-14.2)	4.1 (1.2-14.1)	4.7 (1.3-16.1)	4.4 (1.3-15.5)	3.8 (1.04-13.6)	3.7 (0.99-13.7)
Single/living apart^d^				1.2 (0.2-5.9)	1.3 (0.3-6.6)	1.2 (0.2-6.1)	1.0 (0.2-5.7)	1.0 (0.2-5.7)
Lack of sleep^e^					0.4 (0.1-2.2)	0.3 (0.1-1.9)	0.3 (0.1-2.0)	0.4 (0.1-2.1)
Unemployed^f^						2.2 (0.5-9.8)	2.4 (0.5-10.8)	2.4 (0.5-10.8)
Age^g^							2.8 (0.9-9.1)	2.8 (0.8-9.2)
Multipara^h^								1.1 (0.3-3.6)

^a^EDS ≥13, indicating risk of depression versus not ≤ 13 (reference category).

^b^Low score SOC indicating inability to use their own resources to maintain and improve their health in stressful situations versus medium-high score (reference category).

^c^Miscarriages and abortions versus solely abortion, miscarriages or not at all (reference category).

^d^Single/living apart versus cohabiting (reference category).

^e^Lack of sleep versus adequate sleep (reference category).

^f^Unemployed (including long illness) versus employed (including parental leave and studying) (reference category).

^g^Age ≥35 years versus age 18–34 (reference category).

^h^Parity: primiparae versus multipara (reference category).

## Discussion

This study showed that the prevalence of ‘DV during pregnancy’ was 1%. However, more women reported a history of emotional, physical and sexual abuse performed by their actual intimate partner and also experiences of abuse during the past year. Women born outside Nordic countries were proportionally over-represented among those who experienced ‘DV during pregnancy’. To our knowledge ‘DV during pregnancy’ has not previously been explored among pregnant woman in the same catchment area. Participation recruitment was mostly performed during the first and early second trimesters of pregnancy, and therefore, the results reflect responses to questions about abuse that were posed only once and at this particular time. It has been shown that repeated questioning increases the likelihood of disclosing experiences of physical violence [24,42]. Further, the true prevalence of abuse may be difficult to determine because of fears concerning abuse escalation, if the abuse were to become known by the perpetrator [21]. However, the occurrence of current abuse may also be underestimated due to selection or non-respondent bias. A British longitudinal study indicated that the time of pregnancy was not a sensitive period for DV compared to the postpartum period, where prevalence of physical violence during pregnancy was 1% compared to almost 3% three years later [43]. Therefore, hypothetically, early pregnancy may be protective for women who live in violent relationships. However, the literature is not consistent concerning decreased violence when the woman becomes pregnant [10,16,44,45]. Devries et al. [10] found that in countries reporting high levels of severe IPV, women did not necessarily report high levels of IPV during pregnancy, indicating that cultural factors may be important determinants of IPV during pregnancy. Previous studies have also indicated that IPV could start during pregnancy [45] or be initiated during the first pregnancy [44].

History of physical abuse performed by the actual intimate partner was reported by 2.2% (n = 42) of the subjects. These figures are similar to a previous Swedish study conducted in Uppsala where 2.8% (n = 29) admitted physical abuse by a close acquaintance the year before pregnancy, during pregnancy or 20 weeks postpartum [24]. Nevertheless, it is difficult to compare these results due to the use of different methods and definitions and the lack of separation of history of violence before or after pregnancy from violence during pregnancy. However, in a global perspective the prevalence rates of DV during pregnancy appear to be realistic, since in more developed countries rates seem to be lower than in developing countries [10,11]. However, current results indicate that there is a need for increased attention to this vulnerable group of women who are exposed to violence during their pregnancy and to offer them first line help according to the WHO [46]. It could be of significance for the women exposed to violence to know in what matters society can help them and in what way they can get support from their midwives.

In the present study ‘history of violence’ or lifetime experience of emotional, physical or sexual abuse was reported by 39.5% (n = 761) of the women and was absolutely the strongest indicator of exposure to ‘DV during pregnancy’. This is in accordance with results from a newly conducted meta-analytic review [11]. The response rates reported for ‘history of violence’ are slightly higher than those reported by non-pregnant women visiting gynaecological clinics in Sweden (37.5%) [30], as also measured by the NorAQ instrument. However, the rate reported by Stensson et al. [24] for lifetime emotional, physical or sexual abuse in pregnant women was considerably lower (19.4%), albeit another instrument was used. Also, the Stensson et al. [24] data collection was performed during 1997–1998, and several years have elapsed since this time. The prevalence rates of reported violence against women from the Swedish National Council for Crime Prevention have steadily been increasing during the past years, which partly can be explained by changes in the legislation in the beginning of the 1980s, whereby already submitted written reports about abuse cannot be subsequently withdrawn [26]. Also, media and authorities have called attention to the topic, and hopefully tolerance levels and attitudes towards DV are changing to the benefit of DV survivors.

In the present study the findings showed that the presence of several symptoms of depression was 7.0 fold more likely to be associated with ‘DV during pregnancy’. Those findings are in accordance with a recently conducted meta-analytic review [11]. Both national and international studies show that several symptoms of perinatal depression are indeed significantly associated with the experience of ‘DV during pregnancy’ [16,36,47]. However, the direction of causality with regard to these findings has yet to be determined. The extent to which depression is a consequence of DV or a contributing factor for exposure to DV is entirely unknown. Nevertheless, the most important concern is the pregnant woman’s health, and midwives and other health care professionals need to be aware of these results and to take action accordingly. Screening for depression during pregnancy together with anamnesis on history of violence may be the best way to address DV during pregnancy. The conversation between the pregnant woman and the health care giver must be performed in a safe, confidential atmosphere in an empathic and non-judgmental manner. Both relational ethics, i.e. sensitivity to a specific situation through the initiation of a dialogue between and among individuals [48], and a person-centred care, i.e. an attitude of being with people in a respectful and non-hierarchal way, [49] could be helpful approaches. However, it is not enough to address the violence, but it is also crucial to have guidelines and a plan of action for all health care personnel [50] in an attempt to improve health outcomes for mother and child. However, a recent Cochrane review has presented insufficient data regarding the usefulness of interventions for DV in relation to pregnancy outcomes [51]. Therefore it seems extremely important to focus on testing interventions with the aim of improving the care of those vulnerable women.

### Strength and weaknesses in the study

The strength of the current study is its sample size (n = 1939) and the use of prospectively collected data in a well-defined group of pregnant women. Moreover, the study is only slightly under-powered for detection of prevalence with 98% certainty of DV during pregnancy. However, the results of this study might potentially be biased due to selection or non-respondent bias. Slightly more than 20% of the investigated cohort were women borne outside Sweden. In 2012 approximately 24% of all delivered women in Sweden were foreign borne [52]. These figures suggests that foreign born women are somewhat underrepresented in the material investigated possibly due to language or cultural barriers. Since, proportionally more women born outside Nordic countries reported ‘DV during pregnancy’ suggests the prevalence to be underestimated. Moreover, according to our inclusion criteria participants not understanding Swedish or English were excluded. This might be a weakness with regard to generalisation of the results to the population in the investigated geographical area. In 11 cases the participants did not answer the questions related to violence. Analysis of those 11 women may indicate cultural barriers as there were proportionately more women who did not answer the specific questions about abuse who were foreign born, spoke another language than Swedish at home and had a low level of education. However, it’s also possible, that the questions were felt to be so intrusive that the participant was not prepared to answer them. Only four of the ANC’s receptions have recruited consecutively as instructed and the rest of the receptions have performed convenient recruitment. Therefore, the reported prevalence of current abuse may be underestimated. The data collection period coincided with a strained working situation at the ANCs due to changes in the organization and implementation of a new electronically based medical record system which further increased the work load. An additional possible explanation for under-estimation is that some of the midwives could be an obstacle by themselves. Because of their lack of knowledge about the topic and their fear concerning what to do about disclosure of violence [50], they may have avoided the recruitment of women. Another weakness in the study is uncertainty with regard to exactly how many potentially eligible women were not invited to participate or how many who declined participation in the study. Therefore, unfortunately the prevalence of ‘DV during pregnancy’ may be underestimated. Also, it was not possible to translate the questionnaire to other languages than English, and therefore women who did not have sufficient reading and writing skills in Swedish or English were excluded. However, at least 20% of the included women were foreign-born and originated from 93 different countries.

## Conclusions

The results showed a low prevalence of ‘DV during pregnancy’ in the included group of women from this area of Sweden. However, prevalence rates concerning reported history of emotional, physical and sexual abuse performed by actual intimate partner and history of exposure to violence during the past year indicate that a significant higher prevalence of women are living in a violent relationship. Also, the fact that four of ten women have some ‘history of violence’ which is the strongest factor associated with ‘DV during pregnancy’ must be carefully considered by midwives, obstetricians and other health care givers. Additionally, the knowledge that high levels of depressive symptoms are associated with DV during pregnancy should lead to actions to address mental disorders during early pregnancy. Both ‘history of violence’ and depressive symptoms detected in early pregnancy can indicate that the woman also is exposed to ‘DV during pregnancy’. There is a need to increase attention to this vulnerable group of women who are living in dysfunctional and violent relationships.

## Abbreviations

AAS: Abuse assessment screen; ANC: Antenatal care; AUDIT: Alcohol use disorders identification test; BMI: Body mass index; CI: Confidence intervals; DSM: Diagnostic and statistical manual of mental disorders; DV: Domestic violence; EDS: Edinburgh depression scale; EPDS: Edinburgh postnatal depression scale; IPV: Intimate partner violence; NorAQ: NorVold abuse questionnaire; OR: Odds ratios; SOC-13: Sense of coherence scale-short form; WHO: World Health Organisation.

## Competing interests

The authors declare that they have no competing interests.

## Authors’ contributions

HF and AKD conceived the study. Collection of data were performed by the first author HF. All authors HF, AKD, CWH participated in the study design and coordinated and helped to draft the manuscript. All authors read and approved the final manuscript.

## Pre-publication history

The pre-publication history for this paper can be accessed here:

http://www.biomedcentral.com/1472-6874/14/63/prepub
